# Anticancer Activity of Apaziquone in Oral Cancer Cells and Xenograft Model: Implications for Oral Cancer Therapy

**DOI:** 10.1371/journal.pone.0133735

**Published:** 2015-07-24

**Authors:** Gunjan Srivastava, Raj Thani Somasundaram, Paul G. Walfish, Ranju Ralhan

**Affiliations:** 1 Alex and Simona Shnaider Research Laboratory in Molecular Oncology, Mount Sinai Hospital, Toronto, Canada; 2 Department of Pathology and Laboratory Medicine, Mount Sinai Hospital, Toronto, Canada; 3 Joseph and Mildred Sonshine Family Centre for Head and Neck Diseases, Department of Otolaryngology—Head and Neck Surgery, Mount Sinai Hospital, Toronto, Canada; 4 Department of Medicine, Endocrine Division, Mount Sinai Hospital and University of Toronto, Toronto, Canada; 5 Department of Otolaryngology—Head and Neck Surgery, University of Toronto, Toronto, Canada; School of Medicine, Fu Jen Catholic University, TAIWAN

## Abstract

Oral squamous cell carcinoma (OSCC) patients diagnosed in late stages have limited chemotherapeutic options underscoring the great need for development of new anticancer agents for more effective disease management. We aimed to investigate the anticancer potential of Apaziquone, [EOquin, USAN, E09, 3-hydroxy-5- aziridinyl-1-methyl-2(1H-indole-4,7-dione)–prop-β-en-α-ol], a pro-drug belonging to a class of anti-cancer agents called bioreductive alkylating agents, for OSCC. Apaziquone treatment inhibited cell proliferation and induced apoptosis in OSCC cells *in vitro*. Apaziquone treated OSCC cells showed increased activation of Caspase 9 and Caspase 3, and Poly (ADP ribose) polymerase (PARP) cleavage suggesting induction of apoptosis by apaziquone in oral cancer cells. Importantly, apaziquone treatment significantly reduced oral tumor xenograft volume in immunocompromised NOD/SCID/Crl mice without causing apparent toxicity to normal tissues. In conclusion, our *in vitro* and *in vivo* studies identified and demonstrated the pre-clinical efficacy of Apaziquone, as a potential novel anti-cancer therapeutic candidate for oral cancer management.

## Introduction

Oral squamous cell carcinoma (OSCC) comprise a large proportion of head and neck cancer accounting for an estimated 263,000 new cases and about 127,000 deaths worldwide each year [1]. Early-stage (I and II) OSCC patients are treated with surgery and/or radiotherapy, and have five-year survival rates of 70%– 90% [2–4]. However, two-thirds of OSCC patients suffer from loco-regional advanced disease (stages III and IV) at the time of diagnosis. There exists inadequate data from randomized clinical trials to define an optimal strategy for patients with stages III and IV OSCC. Patients with advanced or recurrent disease have limited treatment options and a poor prognosis (5-year survival rates < 50%) [5]. Primary surgery and definitive radiation therapy are options for OSCC patients; both surgery and radiotherapy can have a profound effect on the quality of life of survivors [6, 7].

In recent years, the application of concurrent chemo-radiation has emerged as an attractive alternative to traditional surgical management of advanced OSCC [8–10]. It is of note that chemotherapy has evolved from palliative care to a central component of curative treatment for locally advanced OSCC. Cisplatin, carboplatin, methotrexate and taxanes are active as single agents or in combination in recurrent or metastatic OSCC [3, 11–14]. However, dose-limiting toxicities in cancer patients restrict their clinical utility. At present, there is no standard second-line chemotherapy regimen for treatment of recurrent or metastatic OSCCs. Monotargeted therapies, such as inhibitors of epidermal growth factor receptor (EGFR), signal transducer and activator of transcription 3 (STAT3), nuclear factor kappa B (NFκB), and Mammalian target of rapamycin (mTOR) have shown limited efficacy [15–18]. Thus there exists a great need for development of new drugs for oral cancer. However, the discovery of new compounds with potent anticancer activity is a long and expensive process. An alternative approach is the exploitation of already established drugs that have been approved for clinical use for other cancers.

Apaziquone [EOquin, USAN, E09, 3-hydroxy-5- aziridinyl-1-methyl-2(1H-indole-4,7-dione)–prop- β-en-α-ol] is a pro-drug belonging to a class of anti-cancer agents called bioreductive alkylaing agents that has undergone extensive clinical evaluation for bladder cancer [19]. Apaziquone is activated by several enzymes, the most widely investigated enzyme being NAD(P)H: quinone oxidoreductase 1 (NQO1) or DT-diaphorase, which reduces apaziquone into a DNA-alkylating agent [19]. Here in we investigated the potential anti-tumor activity of Apaziquone in *in vitro* and *in vivo* models of oral cancer.

## Materials and Methods

### Cell lines and cell cultures

Oral squamous cell carcinoma cell line AMOS III, has been established from betel and tobacco associated human OSCC by our laboratory [20]. AMOS III was used as an *in vitro* and *in vivo* experimental model for oral cancer in this study. Other established OSCC cell line, SCC4, has been used to evaluate the wider applicability of apaziquone for potential oral cancer therapy of OSCC. Non-metastatic oral cancer cell line, SCC4, was obtained from American Type Culture Collection (ATCC). Oral cancer cells (AMOS III/ SCC4) were cultured in Dulbecco’s Modified Eagles Medium (DMEM) containing 10% fetal bovine serum (FBS), 1 mmol/L L-glutamine, and penicillin-streptomycin (1X) in a humidified incubator (5% carbon dioxide, 95% air) at 37°C as described earlier [20–22]. Both the cell lines have been tested using short tandem repeat polymorphism analysis and are being routinely propagated in our laboratory.

### In vitro Cell proliferation/cytotoxicity assay (MTT assay)

The ability of apaziquone to induce cytotoxic effects was determined by the conversion of 3-(4,5-dimethylthiazol-2-yl)-2,5-diphenyltetrazolium bromide (MTT) to formazan by mitochondrial dehydrogenases. Oral cancer cells (AMOS III and SCC4) were plated in triplicates in 96-well plates in complete medium. The cells were cultured to adhere overnight and then exposed to varying concentrations of apaziquone [5 nM to100 μM] for 24 to 96 h to determine dose- and time-dependent inhibition of cell proliferation. Cell proliferation was measured by adding MTT to the cells. Briefly, MTT was dissolved in sterile PBS and added to the wells at a final concentration of 1.5 mM. Cells will be incubated with MTT for 4h, media was removed and the remaining formazan crystals were dissolved in DMSO. The absorbance of solubilized formazan was measured at 540 nm using a multi-well scanning spectrophotometer. The percentage inhibition of cell proliferation was calculated at each time point and dose as follows: (A_control_ − A_treated_/A_control_) × 100.

### In vitro LD_50_ measurements for Apaziquone

To determine the potency of apaziquone to cause cell death of oral cancer cells, its LD_50_ was determined using oral cancer cells AMOS III and SCC4 cells. To determine the concentration of apaziquone required to kill 50% of cells (LD_50_), 5000 cells of each cell line were plated in triplicate in zero fluorescence tissue culture treated 96-well plates (BD Biosciences, Mississuaga, ON, Canada). After overnight incubation, the media was replaced with media containing apaziquone in a concentration range 5nM to100μM. After 48 hours, the alamar Blue assay was performed to determine the cell viability.

### Apoptosis Assay

To verify the results of cell viability assays, Annexin V and propidium iodide (PI) double staining was used to quantify apoptosis. AMOS III cells were either treated with vehicle alone or apaziquone at 500 nM for 48 hours. Cells were labeled with Annexin V–FITC conjugate and PI using the Annexin V assay kit following the manufacturer's instructions (Sigma, St Louis, MO) and analyzed using the BD Cell Quest Pro software. These results were further verified using Western blot analysis for specific caspases and Poly (ADP-ribose) polymerase (PARP) assay.

### Cell cycle analysis using flow cytometry

The cultured media from untreated, vehicle control and apaziquone treated AMOS III cells were collected and centrifuged to collect non-adherent cells. Adherent cells were washed with PBS (pH 7.4) and trypsinized. Both non-adherent and adherent cell populations were pooled for further analysis. Cells were fixed in 70% ethanol (-20°C, overnight) and were resuspended in buffer containing PBS (pH 7.4), EDTA (0.5 mol/L, pH 8.0), Triton X-100 (0.05%), RNase A (50 μg/mL), and PI (100 μg/mL) before flow cytometric analysis.

### TUNEL assay

TUNEL assay was performed following the manufacturer's instructions. Apaziquone-treated and vehicle control AMOS III cells were collected as described above. Labeling and assay was carried out following the manufacturer's instructions, and the results were further analyzed using confocal laser scan microscopy.

### *In vivo* antitumor activity of Apaziquone in xenograft model of human oral squamous cell carcinoma

This *in vivo* study was approved by the Animal Ethics Committee of Mount Sinai Hospital prior to commencement and animal care was done in accordance with the Toronto Centre of Phenogenomics (TCP) guidelines to consider the welfare of the animals and minimize the distress. A preclinical study was performed to compare the efficacy of varying concentrations of apaziquone and vehicle control in an animal model of human oral SCC. The most effective pre-determined concentration/dose of apaziquone was taken forward for *in vivo* testing in xenograft mouse models of oral cancer. Two months old male immune-compromised NOD/SCID/CRL#394 mice were subcutaneously (s.c.) implanted with 1 x 10^6^ cancer cells in serum free medium into the flank region to establish tumors, in compliance with institutional animal care guidelines. When the tumors reached a size of approximately 100–200 mm^3^, the mice were randomized into groups (n = 6/group) according to tumor volumes and body weights for the following treatments: the untreated vehicle control (0.1% DMSO—n = 6) and apaziquone-treated arm of the study (n = 6). In the apaziquone arm of the study, mice bearing the tumors received intraperitoneal instillations of apaziquone at 0.1 mg/kg body weight or vehicle control at 0.1% DMSO 8 weeks after tumor implantation. The drug treatment was continued for 6 weeks. Tumor incidence, tumor volume, weight and overall and median survival of animals were recorded. Mice were monitored twice a week for any signs and symptoms. At the conclusion of the study, blood was collected from the saphenous vein of mice for a complete blood count analysis and organ function test prior to anesthesia. Mice were then euthanized by cervical dislocation or earlier if tumors exceeded 20% body mass and/or if the mice show any clinical signs of paleness, hunched, dyspnea and abnormal movement. Following euthanization, organs were harvested and immediately fixed in formalin. The tumor volume was measured. Tumors were then harvested and an anterior-posterior midline face-cut slice of the tumor was fixed in formalin. The tumor periphery and centre was diced and flash frozen in liquid nitrogen in separate vials. Ki67 immunohistochemistry (IHC) was performed using FFPE tissue sections (4 μm thickness) of tumors from apaziquone treated and vehicle control group mice xenografts following the procedure as described by us earlier [23]. Slides were incubated with anti-Ki67 antibody at a dilution of 1:100 (Abcam, Cedarlane Labs, Burlington, ON, Canada) for 1 h and rabbit secondary antibody for 20 min, followed by VECTASTAIN Elite ABC reagent (Vector labs, Burlingame, CA) using diaminobenzidine as the chromogen. In the negative control tissue sections, the primary antibody was replaced by isotype-specific non-immune mouse/rabbit IgG. The sections were evaluated by light microscopic examination. Hemotoxylin & Eosin (H&E) staining was also performed on paraffin-embedded mouse xenograft tumor sections.

### Statistical analysis

Statistical analysis of the data was carried out using the SPSS 20.0 software (Chicago). Statistical significance was determined using the paired two-tailed Student's t-test. A probability p ≤ 0.05 was considered to be statistically significant. These *in vitro* studies determined the LD_50_ of apaziquone that was used to guide the dose of apaziquone to be used in the *in vivo* studies and establish the mode of cell death in oral cancer cells.

## Results

### Effect of apaziquone on oral cancer cells

**Fig 1:**
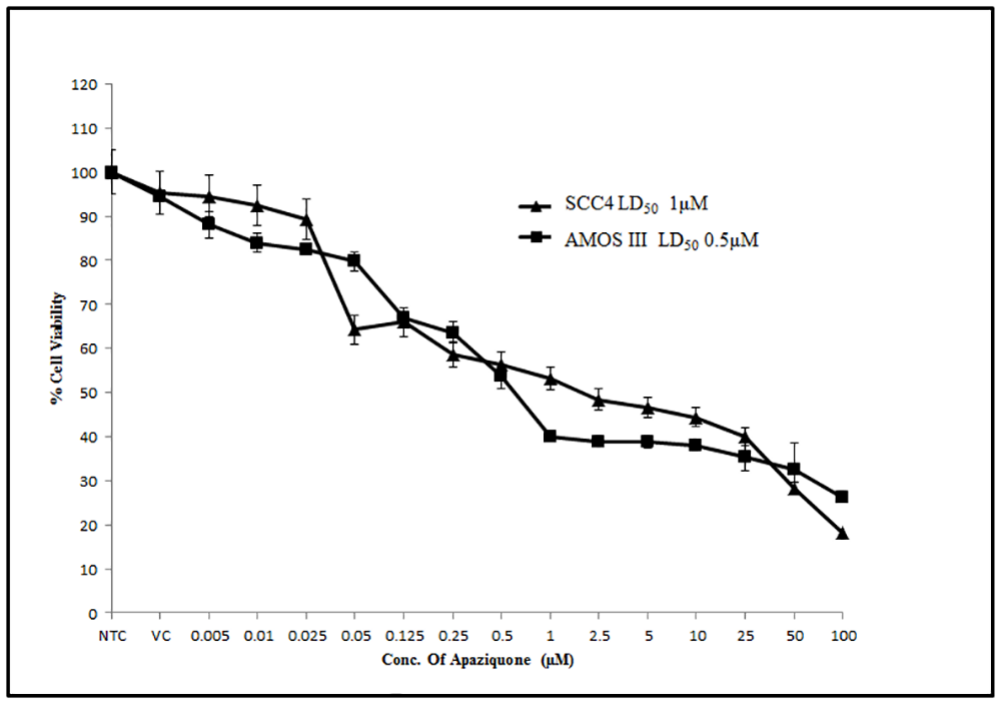
Dose response curve of Apaziquone in oral cancer cells. Alamar blue assay was performed to determine the LD_50_ of apaziquone in AMOS III, and SCC4 cells.

The apaziquone dose response curves in AMOS III and SCC4 cells are shown in Fig 1. The LD_50_ of apaziquone in AMOS III and SCC4 cells were 500nM and 1μM respectively (Fig 1).

### Expression analysis of apoptotic proteins by Western Blotting

**Fig 2:**
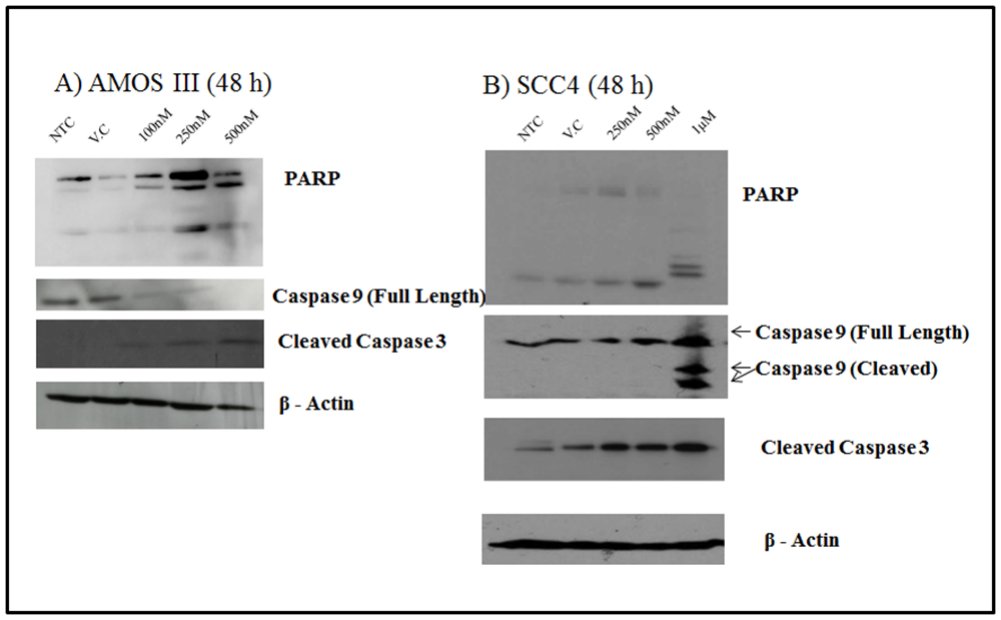
Western Blot analysis of effect of apaziquone on apoptotic proteins in oral cancer cells. Equal amounts of proteins in cell free extracts prepared from AMOS III and SCC4 cells treated with different doses of Apaziquone (250nM, 500nM and 1μM) and untreated as well as vehicle treated control cells were resolved by SDS-PAGE, transferred to PVP membranes, probed with specific antibodies and detected using ECL. Panels show- **(A) AMOS III cells and (B) SCC4 cells.** A dose dependent increase in cleaved PARP, cleaved Caspase 9 and cleaved Caspase 3 was observed in both oral cancer cell lines tested. β-actin was used as a loading control.

Extracted proteins from untreated and apaziquone treated AMOS III cells were analyzed using Western blot for PARP, Caspase 9 and Caspase 3. With increasing dose of Apaziquone expression of the full length Caspase 9 protein decreased and the cleaved Caspase 9 increased, while the expression of cleaved PARP and cleaved Caspase 3 increased. (Fig 2A and S1 Fig) and similar effect of Apaziquone was observed in SCC4 cells on these proteins (Fig 2B and S1 Fig), confirming the induction of apoptosis by apaziquone treatment in both oral cancer cell lines.

### Apaziquone induced apoptotic cell death measured by Tunnel Assay

**Fig 3:**
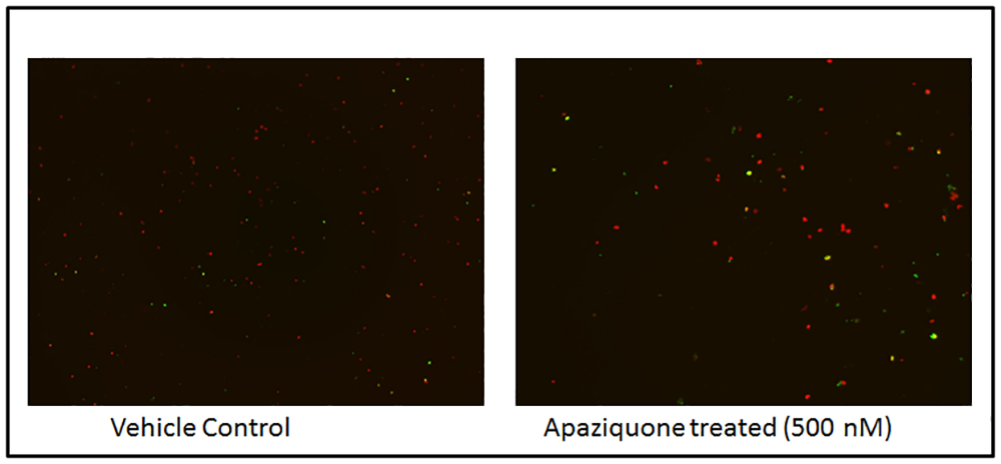
TUNEL assay. Apaziquone treated AMOS III cells showed increased fragments of DNA as compared to the untreated vehicle control cells depicting increased apoptosis in drug treated cells.

Tunnel assay performed on AMOS III cells showed increased DNA fragments in the Apaziquone treated cells as compared to untreated vehicle control treated cells (Fig 3).

### Apaziquone induced apoptotic cell death measured by Annexin V Assay

**Fig 4:**
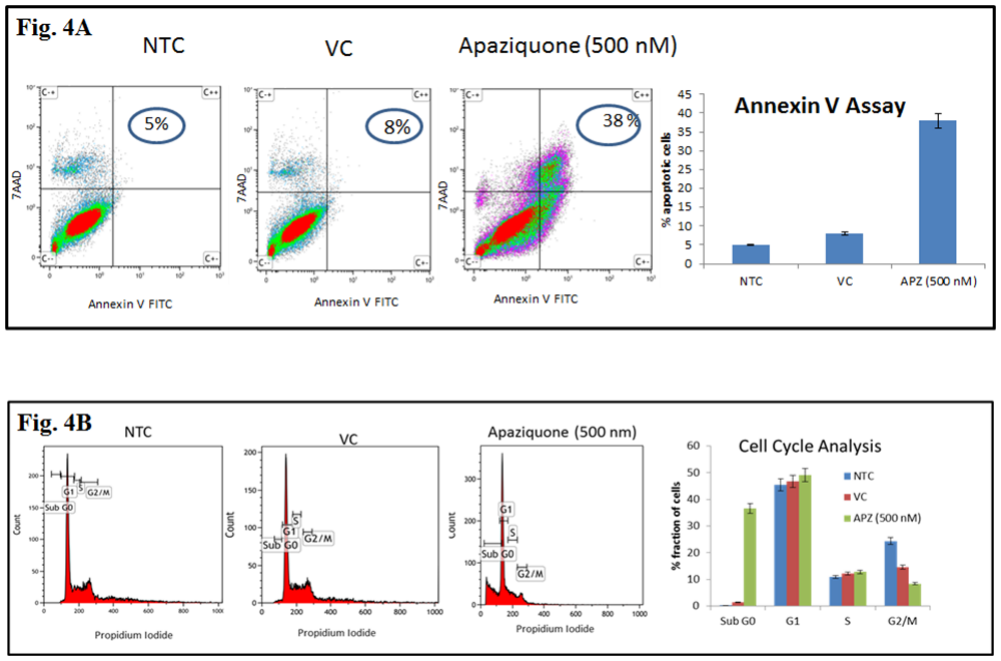
Annexin V assay and Cell Cycle analysis. **(A)** Apaziquone treated AMOS III cells showed significant increase in early and late apoptosis (38%) as compared to the untreated (5%) and vehicle control cells (8%) by annexin V assay. (B) Apaziquone treated AMOS III cells show significantly higher number of cells in the Sub G_o_ phase and G_2_M phases as compared to the untreated control AMOS III cells.

Annexin V assay performed on apaziquone treated AMOS III cells using FACS showed significant increase in early and late apoptosis in Apaziquone treated cells (38%) as compared to the untreated (5%) and vehicle treated control cells (8%) (Fig 4A).

### Apaziquone induced apoptotic cell death measured by Cell Cycle analysis

Cell cycle analysis of apaziquone treated AMOS III cells showed significantly higher number of cells in the Sub G_o_ and G_2_M phases as compared to the untreated control cells confirming increased apoptosis in drug treated cells (Fig 4B).

### *In vivo* anti-tumor activity of apaziquone

**Fig 5:**
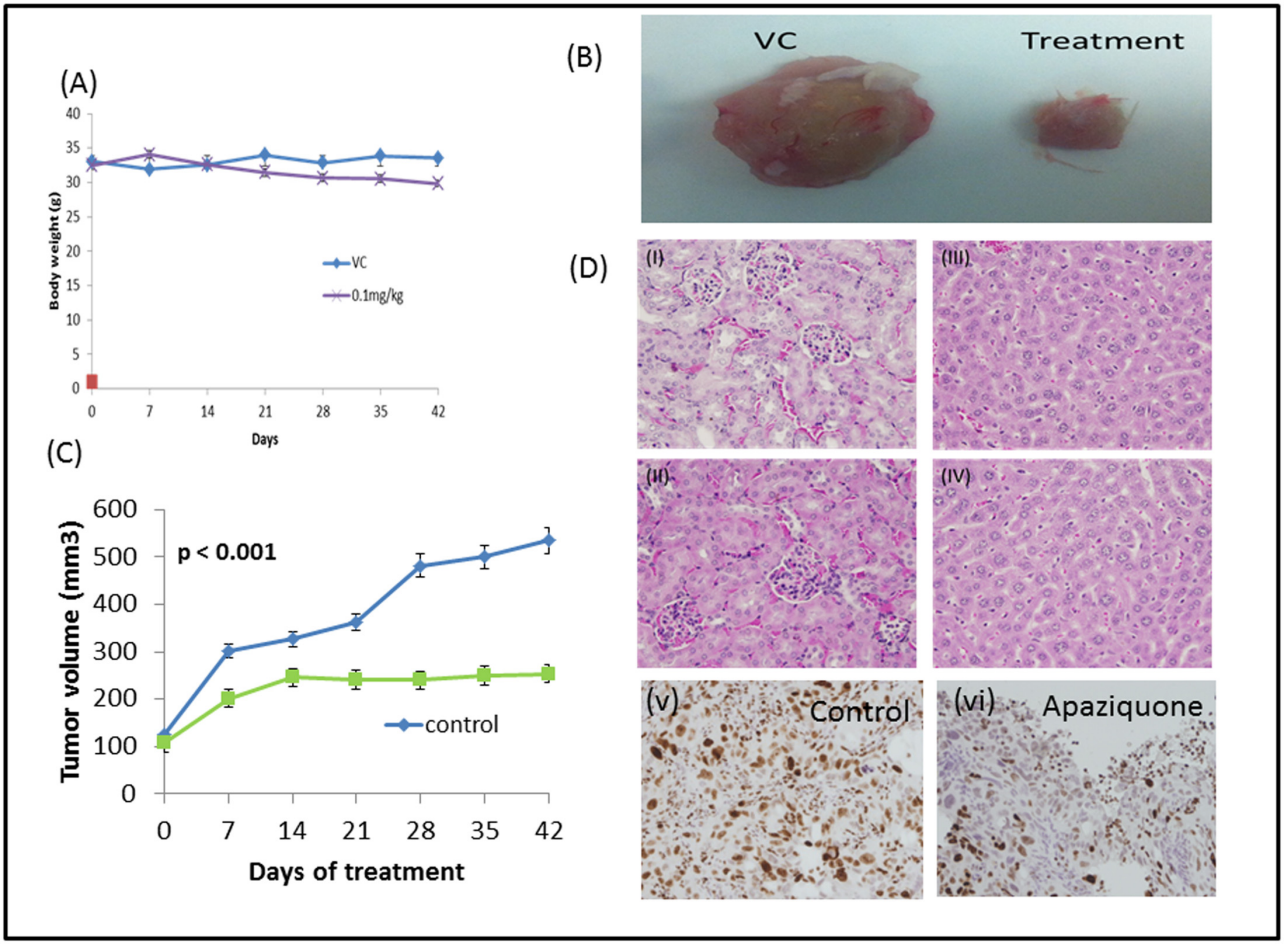
*In vivo* effects of apaziquone treatment. (A) No significant change in body weight was observed in apaziquone treated mice or the vehicle control mice during the course of treatment. (B)Effect of apaziquone treatment on tumor size in mice. Representative excised tumors depicting the difference in the size of tumors between apaziquone treated and the untreated control group mice. (C) Effect of Apaziquone treatment on tumor growth delay. Tumor xenografts developed in the flanks of NOD/SCID/CRL mice were administered with apaziquone (0.1 mg/kg body weight) intraperitoneal injections weekly for 6 weeks. Apaziquone treatment delayed the tumor growth significantly (p value < 0.001, paired two-tailed Student's t-test) in the drug treated group mice as compared to the mice in the untreated control or vehicle control groups. (D) Effect of Apaziquone treatment on kidney and liver of mice. Histology of liver and kidney tissues obtained at the conclusion of the *in vivo* study. Tissue sections were stained with hematoxylin and eosin (H&E). i: Section of liver after the treatment with vehicle control. ii: Section of liver after the treatment with apaziquone. iii: Section of the kidney after the treatment with vehicle control. iv: Section of kidney after the treatment with apaziquone. No oncocytic necrosis or fibrosis was observed in both kidney and liver after the treatment with apaziquone. v: IHC staining for Ki67 of untreated control oral tumor xenograft tissue section showing high nuclear Ki67 immunostaining and vi: apaziquone treated xenograft showing markedly reduced Ki67 staining in the apaziquone treated xenograft tumor tissue section (original magnification x 200).

The *in vivo* antitumor activity of apaziquone was evaluated in oral cancer xenograft models in immune-compromised male mice NOD/SCID/CRL#394. There was no significant change in body weight of the mice in the apaziquone treated group or untreated vehicle control mice over the course of 42 days of treatment (Fig 5A). Tumor growth was significantly delayed by apaziquone (dose 0.1mg/kg) treatment during this time period. This dose was selected from the pilot study which was conducted using multiple doses ranges from 0.05mg/kg to 0.5mg/kg body weight. In the treatment group, the mean tumor volume was 220.05mm^3^ (SD—19.76) as compared to the mean tumor volume of 376.18 mm3 (SD—54.08) in the untreated vehicle control group. This study showed that apaziquone treatment delayed tumor growth significantly (p value<0.001, paired two-tailed Student's t-test between the treated and untreated groups; Fig 5B and 5C).

The apaziquone treated mice showed only few side effects such as minor weight loss, hunched appearance, sunken eyes and unilateral cataract was observed in few mice. However, gross and microscopic (H&E stained) examination of liver showed no obvious signs of oncocytic necrosis or fibrosis in mice in apaziquone treated group. Similarly, no gross or microscopic kidney lesions were observed in mice treated with apaziquone. These results were confirmed by the pathologist (Fig 5D).

Liver function and kidney function tests showed no significant differences between the mice in the apaziquone treated and untreated vehicle control groups. These findings further confirmed there was no toxicity in the apaziquone treated group (S1 Table). However, the complete blood count showed some differences in number of White blood cells (WBC) and neutrophils count. The mice in apaziquone treated group had increased numbers in both WBCs (mean counts 56.1 vs. 33.4) and neutrophils (52.2 vs. 29.7) as compared to mice in the vehicle control group (S2 Table).

## Discussion

The objective of this study was to determine the anti-tumor activity of apaziquone to investigate its potential as an effective alternative to the currently used chemotherapeutic agents for management of OSCC patients. Apaziquone is an indole quinone pro-drug that is activated by cellular DT- diaphorase reductases in well oxygenated regions of the tumors, while under hypoxic conditions apaziquone is activated by Cytochrome P450 reductases [24]. Conversion of apaziquone to active metabolites in turn alkylates the genomic DNA and leads to apoptosis/ cell death. Apaziquone has been shown to be active against different tumor types both *in vitro* and *in vivo* including colon carcinoma, melanoma, renal and non-small cell lung carcinoma and central nervous system cancer cell lines [19, 25]. Apaziquone also showed significant anti-proliferative effects against several murine and human solid tumors, including the generally resistant MAC mouse colon tumors and gastric, ovarian and breast xenografts [26]. Apaziquone has been widely investigated against urinary bladder cancer in not only pre-clinical but multiple clinical studies also [27]. To our knowledge, the current study is the first report on the anticancer effects of apaziquone in oral cancer.

Here in we demonstrated that apaziquone is potently active against oral cancer cells. Our results showed reduced cell proliferation and increased fraction of cells in sub-G_o_-phase of cell cycle suggesting apaziquone induced cell death in OSCC cells. We confirmed apoptosis as major cause for increased cell death in apaziquone treated AMOS III OSCC cells using Annexin V assay. Our findings were further supported by increased levels of fragmented PARP (DNA repair enzyme) following cleavage of caspase 9, suggesting activation of apoptosis in apaziquone treated OSCC cells.

In support of our *in vitro* data demonstrating the efficacy of apaziquone as a novel drug for OSCC, *in vivo* mouse xenograft studies revealed markedly reduced growth of the tumor with minor weight loss in apaziquone treated animals as compared to the vehicle control animal group. The drug treated tumors showed markedly reduced expression of the proliferation marker, Ki67, as compared to the control untreated tumors by immunohistochemistry demonstrating that apaziquone treatment reduced proliferation of tumor cells *in vivo*. Moreover, the sera samples for clinical chemistry profiles, hematology and organ function tests of apaziquone treated mice did not show any apparent toxicity in the treated animals. Taken together, these preclinical findings suggest that apaziquone has a therapeutic effect on oral cancer *in vivo*.

Clinical evaluation of apaziquone was halted by lack of efficacy in phase II trials [25, 28]. Poor drug delivery to tumors caused by a combination of rapid pharmacokinetic elimination and poor penetration through avascular tissue were the major factors responsible for its poor efficacy. Choudry et al [29] suggested that a subset of bladder cancer patients exist whose tumors possess the appropriate biochemical machinery required to activate this drug. Since a significant factor in apaziquone’s failure in the clinic was attributed to its rapid pharmacokinetic elimination resulting in poor drug delivery to tumors, intravesical administration of EO9 was proposed to circumvent the problem of drug delivery to tumors. Based upon an understanding of why apaziquone failed, a further phase I/II clinical trial against superficial bladder cancer using intravesical administration was commissioned in 2001 and sponsored by Spectrum Pharmaceuticals (Irvine, CA) [19]. Significant anti-tumour activity was reported in the phase I/II study [30], and this was subsequently confirmed in phase II studies [31]. Puri *et al* [30] demonstrated that intravesically administered apaziquone is well tolerated locally and systemically, and it has ablative activity for superficial bladder cancer marker lesions. Hendricksen et al., [32] showed that a single immediate post-transurethral bladder tumor resection instillation of apaziquone was well tolerated with an expected good safety profile. Apaziquone and its metabolite EO5a were not detected systemically with pharmacokinetic analyses [26]. Analysis of pooled data from two separate phase III clinical trials for apaziquone in bladder cancer showed significant treatment effect in favor of apaziquone in the primary endpoint of the rate of tumor recurrence at 2 years (p-value = 0.0174) and in a key secondary endpoint, time to recurrence (p-value = 0.0076). However, it did not meet their primary endpoint of a statistically significant difference in the rate of tumor recurrence at 2 years between the two arms [33]. These clinical trials using intravesical administration of apaziquone directly into the bladder supported our rationale for using this drug for oral cancer because oral cavity is readily accessible for localized drug delivery and is likely to improve the pharmacokinetics of apaziquone for clinical use in oral cancer patients.

Loco-regional chemotherapy is emerging as an important adjunct to surgery and systemic chemotherapy in selected patients with some cancers [34–37]. In this context, apaziquone has emerged as a locally efficacious drug in superficial bladder cancer [19]. Our *in vitro* and *in vivo* studies on apaziquone in oral cancer provide preclinical evidence of its efficacy and warrant future phase I and pharmacologic studies in support of its potential clinical use. In the event that systemic administration of apaziquone in oral cancer patients yields limited efficacy, it can be explored for loco-regional chemotherapy as an adjunct to surgery as most sites within the oral cavity are readily amenable to loco-regional drug application.

One of the limitations of our study is that we have not determined the expression level or activity of NAD(P)H quinone oxidoreductase 1 (NQO1) also known as DT-diaphorase 1, the enzyme involved in metabolism of apaziquone in oral SCC cells; in depth investigation of NQO1 in OSCC and its relevance to apaziquone efficacy in OSCC patients will be conducted in a future study. However, NQO1 protein expression has recently been reported to predict poor prognosis of non-small cell lung cancers [38].

## Conclusions

Apaziquone showed promising anticancer activity in oral cancer cell lines killing the cancer cells by apoptosis. Further, apaziquone demonstrated promising anti-tumor activity in oral cancer tumor xenografts without significant toxicity to normal tissues underscoring the pre-clinical efficacy of apaziquone, as a potential novel anti-cancer therapeutic candidate for oral cancer management.

## Supporting Information

S1 Fig Western blot densitometry analysis. Histograms of the western blot densitometry analysis of Caspase 3, Caspase 9, Cleaved Caspase 9, PARP and Cleaved PARP normalized to β-actin in comparison to untreated controls (NTC).
(TIF)

S1 Table Clinical Chemistry Profile, Liver & Kidney function tests. (DOCX)

S2 Table Complete Blood Count. (DOCX)
