# Supplementary figures and images for: Anticancer Activity of Apaziquone in Oral Cancer Cells and Xenograft Model: Implications for Oral Cancer Therapy
Source: PLoS One. 2015 Jul 24;10(7):e0133735. doi: 10.1371/journal.pone.0133735 (PMC4514673; doi:10.1371/journal.pone.0133735)

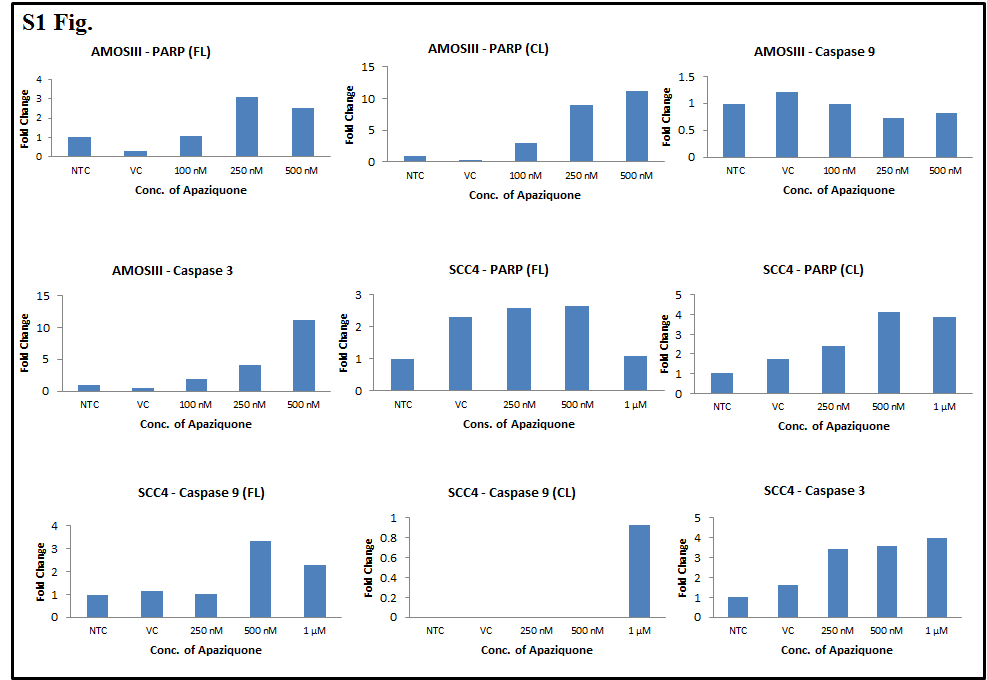

Supplement: S1 Fig — Histograms of the western blot densitometry analysis of Caspase 3, Caspase 9, Cleaved Caspase 9, PARP and Cleaved PARP normalized to β-actin in comparison to untreated controls (NTC). (TIF) [file pone.0133735.s001.tif]
