# Supplementary material for: Anticancer Activity of Apaziquone in Oral Cancer Cells and Xenograft Model: Implications for Oral Cancer Therapy
Source: PLoS One. 2015 Jul 24;10(7):e0133735. doi: 10.1371/journal.pone.0133735 (PMC4514673; doi:10.1371/journal.pone.0133735)
Supplement: S1 Table — (DOCX) [file pone.0133735.s002.docx]

**S1 Table: Clinical Chemistry Profile, Liver & Kidney function tests**

|  | TEST | UNIT | DYNAMIC RANGE | | Vehicle control | | Apaziquone treated | |
| --- | --- | --- | --- | --- | --- | --- | --- | --- |
|  |  |  | Low | High | Mean | SD | Mean | SD |
| ALB | Albumin | g/dL | 1.5 | 6.0 | 2.2 | 0.1 | 2.3 | 0.3 |
| ALP | Alkaline Phosphatase | U/L | 5 | 1500 | 11.0 | 1.0 | 9.8 | 3.5 |
| ALT | Alanine Aminotransferase | U/L | 3.0 | 500.0 | 11.3 | 2.5 | 11.0 | 2.2 |
| AST | Aspartate Aminotransferase | U/L | 3 | 1000 | 47.7 | 9.9 | 56.0 | 7.5 |
| BUN | Urea Nitrogen | mg/dL | 2 | 130 | 18.3 | 0.6 | 20.0 | 5.5 |
| TBIL | Bilirubin, Total | mg/dL | 0.1 | 30.0 | 0.2 | 0.1 | 0.2 | 0.0 |
| CA | Calcium, Total | mg/dL | 4.0 | 18.0 | 9.2 | 0.5 | 9.2 | 0.3 |
| CREAT | Creatinine | mg/dL | 0.2 | 25.0 | 0.2 | 0.0 | 0.2 | 0.0 |
| PHOS | Phosphorus | mg/dL | 1.0 | 20.0 | 6.5 | 0.7 | 6.4 | 0.7 |
| TP | Protein, Total | g/dL | 3.0 | 12.0 | 4.5 | 0.1 | 4.5 | 0.1 |
| B/C | BUN/Creatinine ratio** | - | 0.08 | 650 | 153.3 | 55.1 | 165.0 | 20.8 |
| A/G | Albumin/Globulin ratio** | - | 0 | 100 | 0.7 | 0.6 | 0.9 | 0.6 |
| GLOB | Globulin** | g/dL | 0 | 10.5 | 1.5 | 1.3 | 1.6 | 1.1 |
| NA | Sodium | mEq/L | 50 | 200 | 148.6 | 1.4 | 150.8 | 1.4 |
| K | Potassium | mEq/L | 1.00 | 10.00 | 4.8 | 0.2 | 5.4 | 0.2 |
| CL | Chloride | mEq/L | 50.0 | 200.0 | 106.6 | 0.4 | 108.5 | 0.4 |
| NA/K | Sodium/Potassium ratio** | - | 5 | 200 | 31.0 | 1.4 | 28.0 | 1.4 |
