# Supplementary material for: Anticancer Activity of Apaziquone in Oral Cancer Cells and Xenograft Model: Implications for Oral Cancer Therapy
Source: PLoS One. 2015 Jul 24;10(7):e0133735. doi: 10.1371/journal.pone.0133735 (PMC4514673; doi:10.1371/journal.pone.0133735)
Supplement: S2 Table — (DOCX) [file pone.0133735.s003.docx]

**S2 Table: Complete Blood Count**

| TEST | REFERENCE | | Vehicle control | | Apaziquone treated | |
| --- | --- | --- | --- | --- | --- | --- |
|  |  |  | Mean | SD | Mean | SD |
| WBC (x10^3 cells/µL) | White Blood Cell | 5-12 | 33.4 | 17.2 | 56.1 | 55.0 |
| #NE (x10^3 cells/uL) | Neutrophil Count |  | 29.7 | 16.5 | 52.2 | 53.0 |
| #LY (x10^3 cells/uL) | Lymphocyte Count | 10.5 | 1.6 | 0.5 | 1.4 | 0.7 |
| #MO (x10^3 cells/uL) | Monocyte Count | 0.7 | 0.4 | 0.1 | 0.5 | 0.3 |
| #EO (x10^3 cells/uL) | Eosinophil Count | 0.2 | 0.6 | 0.2 | 0.7 | 0.5 |
| #BA (x10^3 cells/uL) | Basophil Count |  | 0.1 | 0.1 | 0.3 | 0.4 |
| #LUC (x10^3 cells/uL) |  |  | 0.9 | 0.2 | 1.0 | 0.8 |
| % NE | Percent neutrophils |  | 87.9 | 3.4 | 84.7 | 14.2 |
| % LY | Percent lymphocytes |  | 5.3 | 1.9 | 7.8 | 9.1 |
| % MO | Percent monocytes |  | 1.4 | 0.4 | 2.1 | 1.9 |
| % EO | Percent eosinophils |  | 1.9 | 0.3 | 2.8 | 2.9 |
| % BA | Percent basophils |  | 0.2 | 0.1 | 0.3 | 0.2 |
| % LUC | Percent large unstained cells |  | 3.2 | 1.7 | 2.2 | 0.6 |
| RBC (x10^6 cells/µL) | Red Blood Cell | 7-13 | 7.2 | 0.1 | 7.0 | 0.3 |
| HGB (g/dL) | Hemoglobin Concentration | 13-16.9 | 11.6 | 0.5 | 11.7 | 0.9 |
| HCT (%) | Hematocrit | 47.4 | 39.9 | 1.6 | 39.4 | 1.8 |
| MCV (fL) | Mean Corpuscular Volume | 56 | 55.0 | 1.2 | 56.1 | 1.0 |
| MCH (pg) | Mean Corpuscular Hemoglobin | 15.4 | 16.1 | 0.4 | 16.7 | 0.7 |
| MCHC (g/dL) | Mean Corpuscular Hemoglobin Concentration | 27.5 | 29.2 | 0.5 | 29.8 | 0.8 |
| RDW (%) | RBC Distribution Width | 22 | 16.4 | 0.3 | 16.4 | 0.3 |
| PLT (x10^3 cells/uL) | Platelet Count | 350 | 2042.3 | 115.0 | 1804.5 | 342.8 |
| MPV (fL) | Mean Platelet Volume | 351 | 7.2 | 0.3 | 7.8 | 0.3 |
